# miRacle of microRNA-Driven Cancer Nanotherapeutics

**DOI:** 10.3390/cancers14153818

**Published:** 2022-08-06

**Authors:** Goknur Kara, Banu Arun, George A. Calin, Bulent Ozpolat

**Affiliations:** 1 Department of Nanomedicine, Houston Methodist Research Institute, Houston, TX 77030, USA; 2 Department of Chemistry, Biochemistry Division, Ordu University, Ordu 52200, Turkey; 3 Department of Breast Medical Oncology, MD Anderson Cancer Center, The University of Texas, Houston, TX 77030, USA; 4 Department of Translational Molecular Pathology, MD Anderson Cancer Center, The University of Texas, Houston, TX 77030, USA; 5 Houston Methodist Neal Cancer Center, Houston, TX 77030, USA

**Keywords:** miRNA, miRNA mimics, miRNA inhibitors, cancer, nanoparticles

## Abstract

**Simple Summary:**

The discovery of microRNAs has revolutionized the world of science and opened up new opportunities in cancer treatment. miRNA dysregulation plays a crucial role in carcinogenesis processes, such as invasion, metastasis, and angiogenesis, in a broad range of cancers. Although the use of miRNA therapy in cancer treatment is promising, its effective and safe application remains one of the most important challenges hindering its clinical use. Novel nanoparticles continue to be developed and used to enable tumor-targeted miRNA delivery. The aim of the present review is to provide insights into the strategies for miRNA-based therapeutics in cancer, focusing on recent in vivo and clinical studies that have used nanoparticles for miRNA delivery.

**Abstract:**

MicroRNAs (miRNAs) are non-protein-coding RNA molecules 20–25 nucleotides in length that can suppress the expression of genes involved in numerous physiological processes in cells. Accumulating evidence has shown that dysregulation of miRNA expression is related to the pathogenesis of various human diseases and cancers. Thus, stragegies involving either restoring the expression of tumor suppressor miRNAs or inhibiting overexpressed oncogenic miRNAs hold potential for targeted cancer therapies. However, delivery of miRNAs to tumor tissues is a challenging task. Recent advances in nanotechnology have enabled successful tumor-targeted delivery of miRNA therapeutics through newly designed nanoparticle-based carrier systems. As a result, miRNA therapeutics have entered human clinical trials with promising results, and they are expected to accelerate the transition of miRNAs from the bench to the bedside in the next decade. Here, we present recent perspectives and the newest developments, describing several engineered natural and synthetic novel miRNA nanocarrier formulations and their key in vivo applications and clinical trials.

## 1. Introduction

The human genome is known to encode a wide variety of transcripts. The vast majority of RNAs do not code for protein and are termed non-coding RNAs (ncRNAs) [1,2]. miRNAs bind to target messenger RNA (mRNA), leading to silencing of the target mRNA/gene expression through translational repression or degradation of mRNA [3]. Most miRNAs are preserved in animal species, indicating the evolutionary significance of these molecules as regulators of vital cellular activities, such as proliferation, differentiation, apoptosis, and metabolism [4,5,6,7]. Aberrant expression of miRNAs and/or disruption of their function play important roles in the development of various pathological conditions, including cancer [8]. The dysregulation of miRNA expression leads to improper control of the functions of a wide number of proteins and biological mechanisms impacting cancer-associated signaling processes [9,10].

Given the ability of miRNAs to modulate multiple oncogenic mRNAs and related oncogenic pathways, miRNA therapy offers an effective strategy for the development of targeted cancer therapeutics. In general, there are two main approaches using two types of miRNA-based therapeutics regarding oncological pathologies [11]: (1) restoration of reduced/lost tumor suppressor miRNA expression through delivery into tumor tissues (miRNA mimic therapy) and (2) inhibition of oncogenic miRNA through the use of antisense oligonucleotides/antagoMirs/antimiRs (miRNA inhibition therapy) [8,12,13,14,15].

Despite remarkable advances in miRNA-based therapeutics, naked miRNA molecules present various challenges in terms of efficacy, drug design, and clinical use [16]. These challenges prevent naked miRNAs from safely reaching target cells or tissues and starting their activity when they are introduced into circulation. In the last decade, considerable efforts have been devoted to overcoming the issues arising from miRNA delivery. Nanotechnology-based carrier systems have been developed as effective, suitable, and safe miRNA delivery vectors [17]. Nanodelivery systems can be designed to encapsulate negatively charged miRNAs via electrostatic interactions, thereby protecting miRNAs from degradation, and safely deliver miRNAs to tumor tissues to hit the designated genes in the target cell. Additionally, ideal nanocarriers are expected to be non-toxic and have high miRNA binding capacities, selectively release miRNA, and allow for easy surface functionalization [18,19].

In this review, we briefly describe the principles of the miRNA biogenesis process and the role of miRNAs in cancer. We then discuss the miRNA-based therapeutic strategies, their limitations, and the advantages of miRNA delivery when using a nanocarrier over just naked miRNA. Finally, we highlight in detail the recent developments in the design of novel nanoformulations and the clinical outcomes achieved using nanocarrier-mediated miRNA therapy in cancer.

## 2. Biogenesis of microRNA

**Figure 1:**
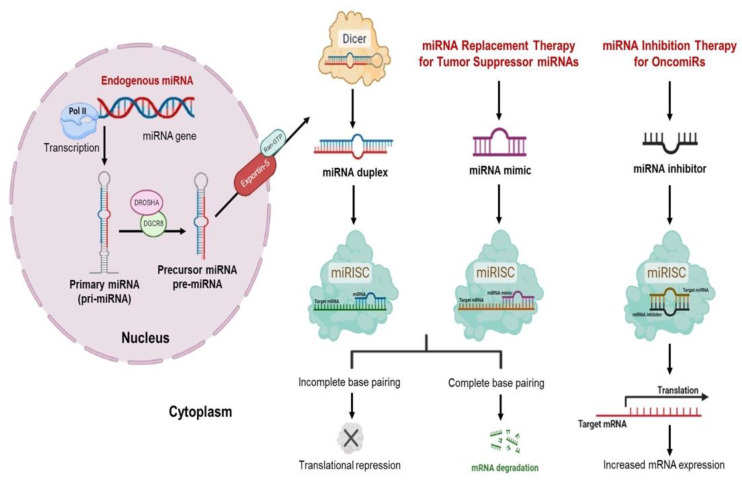
miRNA biogenesis, replacement of tumor suppressor miRNAs, and inhibition of miRNA function by miRNA inhibitors. In the nucleus, the miRNA gene is transcribed by RNA polymerase II into the pri-miRNA, which is then cleaved into the pre-miRNA by Drosha and DGCR8 activation. The pre-miRNA is exported to the cytoplasm by exportin 5 and further processed by Dicer into a mature miRNA duplex. The miRNA is uploaded to the RISC-AGO to target mRNA, resulting in translational repression or the degradation of the target mRNA. Synthetic double-stranded miRNA mimics can be used to restore a specific tumor suppressor miRNA in miRNA replacement therapy, leading to translational repression or the degradation of all of its mRNA targets. Oncogenic miRNA function can be inhibited by miRNA inhibitors, which bind to target endogenous miRNAs, leading to increased mRNA expression of the tumor suppressor gene.

miRNA genes are located within the genome as monocistronic (single miRNA genes), polycistronic (clusters), or intronic (in introns of protein-coding genes) miRNAs [20]. Genomic studies suggest that miRNA transcription is closely coordinated with the transcription of the protein-coding genes that are targeted by miRNAs [21,22]. Canonical miRNA biogenesis is initiated with the transcription of endogenous miRNA genes into long primary miRNAs (pri-miRNAs) by RNA polymerase II (Pol II) [23]. Typically, these pri-miRNAs are comprised of a stem region (33–35 base pairs), a terminal loop structure, and one single-stranded RNA (ssRNA) segment located in the 5′ and 3′ end, respectively. The mature miRNA sequence of 20–25 nucleotides is embedded in the stem region [21,24]. The nuclear microprocessor complex, consisting of the RNase III-type enzyme Drosha and the double-stranded RNA (dsRNA)-binding protein (dsRBP) DiGeorge syndrome critical region 8 (DGCR8), catalyzes pri-miRNA processing. After DGCR8 recognizes and binds to the stem of the pri-miRNA, Drosha cleaves both ssRNA flanking segments and the stem sites of ~11 base pairs of the pri-miRNA, yielding the precursor miRNA (pre-miRNA) [22,25,26]. Pre-miRNAs are hairpin RNA structures ~65–70 nucleotides in length with two nucleotide overhangs at the 3′ end [27]. Once pre-miRNAs are exported to the cytoplasm by activation of exportin-5 protein (XPO5) and its cofactor Ran-GTP, the RNAse III-type enzyme endonuclease Dicer processes the stems of the pre-miRNAs into a 20–25 nucleotide long miRNA duplex [28,29]. Both strands of the miRNA duplex are then loaded into one of the proteins of the Argonaute (AGO) family to form the RNA-induced silencing complex (RISC). AGO protein is responsible for selecting one of the strands of the miRNA as the guide strand of the mature miRNA and removing the remaining strand, which is called the passenger strand [30]. Conventionally, the -5p and -3p suffixes are added to pinpoint the mature miRNAs derived from the 5p and 3p miRNA duplexes, respectively; however, some miRNAs are known to have both strands active [31]. The complex (miRISC), consisting of the mature miRNA, AGO, and the RISC, interacts with the target mRNA and binds to a specific sequence at its 3′ untranslated region (3′UTR). This generally results in translational repression or degradation of the target mRNA, depending on the complementarity degree with the target mRNA sequences [32,33]. The complete base pairing of the miRNA with the target mRNA results in mRNA degradation, while incomplete base pairing promotes protein translational repression [34]. The target recognition occurs between the seed sequence of the miRNA (nucleotides 2–7) and the target mRNA. Single miRNA may interact with or suppress hundreds of different target mRNAs if its seed sequence is too short, adding to the complexity of post-transcriptional regulation [20,35]. Therefore, miRNAs are considered master regulators of cellular functions, as they play a significant role in nearly all the molecular pathways in both physiological and pathological processes [36]. miRNA biogenesis is represented in Figure 1.

## 3. MicroRNAs and Cancer

Approximately 2694 human mature miRNAs have been identified [37] (miRBase Release 22: http://www.mirbase.org, accessed on 6 March 2018), and 60% of all human mRNA genes have been estimated to be regulated by miRNAs [38]. Alterations in miRNA result in adaptative and fast changes in gene expression and have a remarkable influence on the development of multiple human diseases [36], such as neurodegenerative [39] and cardiovascular [40] disorders, diabetes [41], and cancer [42].

Cancer is considered the most important disease in terms of miRNA–mRNA interactions, as severe dysregulation of miRNAs in various cancers have been discovered over the last two decades. A genome-wide analysis profiling 186 miRNAs revealed that miRNA genes are frequently located at fragile regions or common cancer-related genomic sites in humans [43]. Almost all human cancers have alterations in miRNA expression, which can be detected by advanced high-performance technologies, such as expression microarrays, gene chips, or next-generation sequencing techniques [44,45]. Genetic aberrations, changes in transcriptional regulation, and malfunctions in miRNA biogenesis have been characterized in numerous tumor types; these aberrations result in overexpression or reduced/lost expression levels in miRNAs [46]. miRNA expression profiles of tumor tissues in comparison to the corresponding normal tissues indicated that altered miRNA expression is highly correlated with the tumor origin and its progression patterns [47]. Therefore, aberrantly expressed miRNAs are expected to hold therapeutic targeting, diagnostic, and prognostic implications [48].

miRNAs’ relevance to cancer was first demonstrated by George A. Calin and his colleagues in 2002 [49]. They discovered that miRNA cluster miR-15a–miR-16-1 expression was lost or downregulated in 69% of B-cell chronic lymphocytic leukemia (B-CLL) patients and corresponded to the loss of the 13q14 chromosomal region [49]. Further studies demonstrated that these miRNAs have a tumor-suppressive role and target/repress the antiapoptotic proteins, including Bcl-2 and Mcl-1 [50]. Besides their tumor suppressor function, the oncogenic activity of miRNAs was first established in 2005 by He et al. [51]. Overexpression of the miR-17/92 cluster, which is highly expressed in B-CLL patients, was found to promote lymphoma development in vivo through induction of the c-Myc oncogene [51]. Later studies demonstrated that miRNAs are involved in carcinogenesis, tumor growth, and progression by modulating several processes, including cell proliferation, invasion, migration, and apoptosis [52,53]. Dysregulation in miRNA expression in tumors results in either downregulation of tumor suppressor miRNAs or upregulation of oncogenic miRNAs [53]. Considering the critical role of miRNAs in tumorigenesis and their capacity to regulate oncogenes and related downstream pathways simultaneously, increasing attention has been given to using miRNA-based therapeutics in cancer in recent years [54].

## 4. miRNA-Based Therapeutic Strategies in Cancer

There are two miRNA-based therapeutic approaches: (1) restoration of reduced or lost tumor suppressor miRNA levels in cancer cells by delivering miRNA mimics directly to cancer cells to enhance the expression of particular tumor suppressor miRNAs, and (2) inhibition of highly overexpressed oncogenic miRNAs using miRNA inhibitors, such as antagomiRs, antisense oligonucleotides, or small molecule inhibitors, to reduce abnormal miRNA levels [55,56]. miRNA-based therapeutic strategies for cancer are represented in Figure 1.

### 4.1. miRNA Mimic Therapy

This approach involves restoring reduced/lost tumor suppressor miRNAs that have an inhibitory effect on cell proliferation, invasion, and angiogenesis, altogether affecting tumor growth and progression by repressing the translation of tumor-promoting or oncogenic genes/mRNAs [54]. Resulting from epigenetic silencing, genomic mutation or deletion, or changes in miRNA mechanism, the expression of such miRNAs tends to be lost or downregulated in tumors [47]. Since such reduced/lost miRNA expression contributes to oncogenic activation, the expression of therapeutic miRNA mimics can suppress tumor-promoting genes using exogenously introduced miRNA. miRNA mimics are oligonucleotides that are chemically synthesized as double-stranded RNA duplexes [57,58]. They have a guide strand, the sequence of which is the same as the mature miRNA, that imitates the function of the endogenous miRNA when introduced into cells. The other strand, which is partially or entirely complementary to the guide chain, is called the “passenger strand” [55]. miRNA mimics participate in the miRNA biogenesis machinery in the cytoplasm and are loaded into the RISC complex, which further leads to translational repression or degradation of the target mRNA [54] (Figure 1).

### 4.2. Anti-oncomiR Therapy

miRNAs with oncogenic effects (oncomiRs) are abundantly expressed in a variety of cancers owing to their transcriptional dysregulation via epigenetic mechanisms or gene amplification [59]. These oncomiRs promote tumor growth and progression by negatively inhibiting tumor suppressor genes and/or genes that play an important role in apoptosis, cell differentiation, and cell cycle progression [60,61]. OncomiRs can be therapeutically targeted for inhibition, thereby ensuring the restoration of the normal function of target tumor suppressor genes [62]. This targeting approach includes miRNA inhibitors, which are single-stranded oligonucleotides able to bind a specific endogenous miRNA via their sequence complementarity and, consequently, eliminate these miRNAs from the RISC complex [54]. Thus, targeted inhibition of a specific miRNA followed by upregulation of its target mRNAs can be achieved [54]. In recent years, anti-miRNA oligonucleotides (AMOs) [63], antagomiRs [64], locked nucleic acids (LNAs) [65], and miRNA sponges [66] have been described as commonly used miRNA inhibitory approaches.

## 5. Delivery of miRNA-Based Therapeutics into Cancer Cells

Despite the fact that several miRNA-based therapeutics, either miRNA mimics or anti-miRs, have recently entered human phase 1/2 clinical trials, their transition from the bench to the clinic has been slow and very challenging [67]. The major obstacle to the translation of miRNA therapeutics into the clinic has been identifying methods for safe delivery of miRNA mimics or inhibitors that effectively target tumor tissues [34,68]. The half-lives and biodistribution of naked miRNAs in the circulatory system are very short and poor, respectively. Therefore, naked miRNAs are easily degraded, within seconds, by the abundant nucleases present in the bloodstream and eliminated through renal and hepatic clearance, leading to limited in vivo stability [69,70,71]. Due to their intrinsic negative charge and hydrophilic structure, intracellular uptake of miRNAs is difficult [72,73]. However, even if they get inside a cell, these naked miRNAs are prone to rapid endolysosomal degradation [52]. Activation of the immune system by secretion of inflammatory cytokines and type I interferons (IFNs) leads to critical toxicological consequences after systemic administration of miRNA therapeutics [68]. Another concern in miRNA delivery arises from the potential off-target effects. Since miRNAs may partially bind the 3′UTR of other genes, they may induce an undesired off-target effect that may lead to adverse events [74]. Strategies developed to overcome this problem include chemically modifying the exogenous miRNA at the 2′OH group [75], using modified LNAs [76], redesigning the passenger strand [77], and using peptide nucleic acid (PNA) complexes [78]. Even though chemically modified miRNAs have been reported to enhance in vivo delivery and stability by reducing immunogenicity and sensitivity to nucleases, they remain incapable of addressing the existing challenges related to poor cellular uptake, toxicity, and off-target effects. Therefore, to address these hurdles, viral and non-viral vectors have been developed for the delivery of modified or unmodified miRNA therapeutics [79]. Viral vectors, such as adenoviruses, adeno-associated viruses, or lentiviruses, could be able to deliver miRNA modulators to target cells with high transduction capabilities; however, their application is limited due to their immunogenic concerns [80]. Thus, non-viral vectors can be considered safer and as having fewer side effects, provided that their cell integration efficiency can be greatly improved to achieve successful and sustained miRNA cell delivery. Recent successful reports on effective nanocarrier systems delivering miRNA-based therapeutics in human phase 1 and 2 clinical trials have generated significant excitement in the field [81].

## 6. Nanocarrier Systems to Deliver miRNA-Based Therapeutics

Nanocarriers are made of diverse materials, such as polymers, lipids, iron oxide, gold, etc., with particle diameters ranging from 1 to 1000 nm. The ideal nanoparticles have specific properties, including biocompatibility, biodegradability, low immunogenicity, increased cellular uptake, endosomal escape, selective accumulation at the tumor site, cost-effectiveness, and simple fabrication [74,82,83,84]. The benefits of using nanocarrier systems include protecting the payload’s stability and increasing its half-life in circulation [85]. Moreover, nanodelivery systems can be modified with specific tumor-targeting ligands for cell surface receptors to achieve active targeting [86]. In this review, we focus on the recent developments and applications of novel nanocarriers for the delivery of miRNA-based therapeutics. Table 1 demonstrates the nanoparticles recently used for the in vivo delivery of miRNA mimics and inhibitors.

### 6.1. Polymer-Based Nanocarriers for miRNA

The use of chemically modifiable polymers as nanocarriers for miRNA delivery has been comprehensively explored due to their low toxicity and their high versatility [3]. Negatively charged phosphate groups of miRNAs interact with positively charged functional groups of these nanosystems through electrostatic interactions, leading to the formation of polyplexes [87]. The simplicity of manufacturing techniques and the cost-effectiveness of the raw materials make them favorable for industrial scale-up [88]. In general, polymers can be classified natural and synthetic. Proteins, peptides, and polysaccharides are classed natural polymers, whereas polyethyleneimine (PEI), poly(lactic-co-glycolic acid) (PLGA), poly-L-lysine (PLL), poly-L-arginine (PLA), and dendrimer are the main synthetic polymers.

#### 6.1.1. Natural Polymer-Based Nanocarriers

##### Protein-Based Nanocarriers

Recently, nanocarriers composed of proteins (albumin, sericin, fibroin, gelatin, and atelocollagen) have gained attention, as they are non-toxic, non-immunogenic, biodegradable, and highly biocompatible and involve materials that are easy to prepare, with enhanced blood circulation times and long shelf lives [89,90,91]. The abundance of hydroxyl, amino, and carboxyl groups in their structures enables them to bind to a variety of ligands, drugs, oligonucleotides, or RNA molecules, thus providing various surface interactions [92].

*Albumin* is the major serum protein made by the liver that accounts for 55% of all blood proteins and transports serum lipids and steroid hormones. Due to its non-toxic, non-immunogenic, biocompatible, and biodegradable features, albumin has been used in preparing nanocarriers to improve the delivery of antisense oligonucleotide [93,94] and siRNA [95] therapeutics with impressive results. Since albumin molecules contain a large amount of negatively charged amino acids, such as aspartate and glutamate, and positively charged amino acids, such as lysine and arginine, albumin-based nanocarriers are favorable for electrostatic adsorption of cationic or anionic molecules. Albumin-based nanocarriers tend to accumulate in the tumor site through a specific transendothelial transport process, increasing the tumor-targeted delivery potential [96,97]. Silk sericin is a highly hydrophilic biopolymer and natural protein found in silk fibers together with fibroin [98]. Due to its rich amino acid content and polar chemical groups, sericin can be mixed with other polymers via easy cross-linking, forming more advanced biomaterial-based nanoparticles with superior properties [99,100]. However, only a small number of reports on DNA [101] and siRNA [102] delivery applications of sericin-based nanocarriers are currently available. Despite their great potential, there is still a lack of research on albumin- or sericin-based nanocarrier systems for the delivery of RNA therapeutics and, in particular, miRNAs. It was in this context that we previously reported a novel nanocarrier design composed of a blend of albumin and silk sericin for in vivo miRNA delivery [103]. We found that systemic injection of miR-329 mimic incorporated into albumin-sericin nanocarriers at therapeutic doses resulted in significant in vivo tumor integration of miR-329 and inhibition of the desired target genes in multiple triple-negative breast cancer (TNBC) models. Specifically, we documented significant downregulation of the eukaryotic elongation factor-2 kinase (eEF2K) protein in tumors with marked anti-tumor efficacy. Moreover, we did not observe any side effects during four weeks of treatment in mice, suggesting that albumin-sericin-based miR-329 mimic nanotherapy is safe and effective, with great potential for clinical application [103].

*Gelatin* is another protein that has been utilized to generate colloidal drugs or gene nanodelivery systems for biomedical and pharmaceutical applications. It is a natural biopolymer obtained from acid or alkaline hydrolysis of animal collagen [104,105], and it also has highly biocompatible, biodegradable, non-toxic, and non-immunogenic features. There are several studies on gelatin-based sustained- and controlled-release delivery systems for miRNA therapeutics. Liu et al. [106] demonstrated for the first time that a gelatin-based nanosphere delivery system incorporating miR-506 mimic led to a marked inhibition of tumor growth and metastasis in TNBC xenograft models in mice.

*Atelocollagen* is a natural, soluble fibrous collagen with an important role in maintaining the morphology of tissues and organs that has emerged as a potential gene delivery [107]. Hao et al. [108] developed atelocollagen (ATE) miRNA nanoparticles coated with an RNA aptamer (APT) ligand that target prostate-specific membrane antigen (PSMA). This nanocarrier system was able to provide selective and enhanced delivery of tumor-suppressive miRNAs, such as miR-15a and miR-16-1, into PSMA-positive prostate cancers in vitro and in vivo.

##### Peptide-Based Nanocarriers

Cationic peptides, such as protamine, PLL, and cell-penetrating peptides (CPP), have been extensively studied for RNA delivery owing to their condensation ability with negatively charged RNA, ease of manufacte, and the adjustable size of the nanoparticles [109].

*Protamine* is a highly positively charged, naturally occurring polypeptide consisting of abundant amounts of arginine [109,110]. Protamin is classified under the umbrella of the FDA-approved drug heparin. In a recent study, novel protamine nanocapsules were used for delivery of miR-145 mimic in colorectal cancer models [111]. For this study, investigators used protamine nanocapsules that were first loaded with miR-145 and then coated with an external layer of protamine for incorporation of curcumin for added functionality.

*Tachyplesin* (*Tpl*) was developed as a novel nanocarrier to deliver anti-miR-210, as miR-210 is a highly expressed oncogenic miRNA in glioblastoma (GBM) [112]. Tpl-anti-miR-210 led to a significant inhibition in miR-210 expression level of up to ~90% and reduced the cell proliferation, migration, and spheroid-formation capacity of GBM cells. In another study, novel chimeric peptides consisting of plectin-1-targeting peptides (PTP) and arginine residues were generated to specifically deliver miR-212 mimic into pancreatic ductal adenocarcinoma (PDAC) cells [113]. Researchers obtained spherical nanoparticles in sizes ranging from 100 to 200 nm after mixing plectin-1 (PL-1) and miR-212. They also reported that PL-1/miR-212 nanoparticles markedly increased the response to chemotherapeutics, such as doxorubicin (DOX), in both in vitro and in vivo pancreatic cancer patient-derived xenograft (PDX) models of PDAC cells. The same researchers also utilized these nanoparticles for the delivery of miR-9 mimic in vitro and in PDX models [114]. Similarly, the combined treatment of DOX with PL-1/miR-9 nanocomplexes dramatically enhanced the anti-tumor impact of DOX, which was accompanied by an inhibition of the expression of the target gene eIF5A2 and induction of apoptosis in in vivo PDAC models.

##### Polysaccharide-Based Nanocarriers

Natural polysaccharides, including chitosan, dextran, hyaluronic acid, cyclodextrin, and their derivatives, have been widely used as backbone materials in developing nanocarriers for miRNA delivery [115]. These materials are considered very promising for the preparation of nanosized carriers due to their outstanding characteristics, such as low toxicity and immunogenicity, high biodegradability, biocompatibility, stability and ease of modification, cost-effectiveness, and abundant availability [116]. Most polysaccharides and polysaccharide derivatives possess hydrophilic groups, such as hydroxyl, carboxyl, and amino groups [117]. Thus, polysaccharides can overcome the limitations in the clinical translation of many drugs, including miRNA therapeutics [118].

*Chitosan*, a native cationic polysaccharide, has been broadly and successfully utilized for gene transfection for decades due to its primary amine groups, providing a positive charge at acidic pH and enabling complexation with polyanions A, such as DNA [119], siRNA [120,121], and miRNA [122] oligonucleotides. However, the low solubility of chitosan molecules at physiological pH reduces nanoparticle stability in the circulatory system, transfection efficiency, and RNA activity and creates issues with scaling up, entailing limitations for future in vivo clinical applications. Therefore, various modifications of chitosan are preferred, since it is soluble only at acidic pH levels [123]. In a recent study, Amgoth et al. [124] generated copolymer-based nanoparticles (NPs) using chitosan (C) as a backbone and L-arginine (L-Arg) as lateral chains and then decorated this copolymer with gold nanoparticles (AuNPs), resulting in nest-type perforated C-A-Au NPs. These nanoparticles were ∼150 nm in diameter and highly cationic, with a greater loading capacity for gefitinib (GFT) and miR-125b. C-A-Au NPs incorporating GFT-miR125b preferably accumulated at the tumor area in the Lewis lung carcinoma (LLC) xenograft tumor model and inhibited EGFR signaling, leading to tumor growth inhibition. A novel nanocarrier, hyaluronic acid (HA)-modified and conjugated linoleic acid (CLA)-grafted chitosan nanoparticles co-loaded with miR-34a and DOX, has been formulated [125]. Therapeutic administration of these nanosystems resulted in 73.7% tumor inhibition of MCF-7/A tumor xenografts in mice, which was higher than the other treatment arms lacking the targeting ligand, the miR-34a, or DOX. Chitosan-based nanoparticle delivery of miR-34a, a tumor-suppressive miRNA that downregulates multiple gene products involved in prostate cancer progression and metastasis, inhibited tumor growth and preserved bone integrity in a xenograft model representative of established prostate cancer bone metastasis [126].

*Dextran* is an FDA-approved branched microbial polysaccharide that has found a broad range of uses in drug conjugation approaches based on its desirable features, including reduced toxicity, bioavailability, biodegradability, and low cost, along with its long history of clinical usage [127]. It is a highly stable and hydrophilic polymer that has also been used in nanomedicine [128]. Various dextran-based nanocarriers with rational modifications, such as PEI-dextran-coated iron oxide nanoparticles [129], lipid-modified dextran-based polymeric nanoparticles [130], carboxymethyl dextran-stabilized PEI-PCL (poly(epsilon-caprolactone)) nanoparticles, and chitosan-thiolated dextran nanoparticles [127], have been used to deliver several miRNA therapeutics. In an interesting work conducted by Zheng et al. [131], spermine-modified acetalated dextran nanoparticles (SpAcDex NPs) were synthesized, modified with bradykinin ligand agonist targeting B1 receptor, and encapsulated with anti-miR-21, aiming to upregulate PTEN for the treatment of glioblastoma multiforme (GBM). These B1L-targeted SpAcDex NPs integrated with tumors efficiently by traversing the blood–tumor barrier (BTB), upregulated PTEN expression, and caused a powerful tumor anti-angiogenic effect in experimental GBM models.

*Cyclodextrins* are biocompatible oligosaccharides obtained from starch. This polymer was first described as a plasmid DNA delivery agent; thereafter, cyclodextrin-based nanomaterials were widely studied for the development of siRNA delivery systems [132,133,134]. They have a hydrophobic inner side and hydrophilic outer surface with -OH groups that allow for modification with cationic or amphiphilic polymers and enable targeting agents to achieve improved gene delivery efficacy [135]. Owing to their unique chemistry, these compounds have been strongly considered for cancer therapy [136]. CALAA-01 is the first targeted siRNA drug that contains cationic cyclodextrin-based polymeric nanoparticles as carrier components [137]. Recently, cyclodextrin-based nanostructures have emerged as promising candidates for miRNA delivery in cancer. Shao et al. [138] fabricated a polymeric nanoconstruction based on the self-assembly interaction of PEI-crosslinked β-cyclodextrin (PEI-βCD) and functional adamantly (Ad) moieties named poly(ethylene glycol) (PEG). CDM was added as a pH-responsive linker to increase the function of the therapeutic miRNA. These miRNA-loaded nanoparticles were found to have an average size of around 150 nm and a zeta potential of 20 mV; miR-199a/b-3p mimic and anti-miR-10b were co-encapsulated in these nanoparticles for simultaneous delivery to target cells. The sucessful tumor cell integration of these multi-functionalized nanoparticles proved to drastically suppress tumorigenesis by targeting mTOR, PAK4, RHOC, and the epithelial–mesenchymal transition (EMT) in hepatocellular carcinoma (HCC) PDX models. The synergistic efficacy of cyclodextrin-based cationic star co-polymer (sCDP) nanocarrier-mediated miRNA therapy and chemotherapy was investigated in hepatoma treatment by Xiong et al. in 2021 [139]. While the hydrophobic drug DOX was loaded to the inner core of sCDP, the DOX/sCDP self-assembled into nanostructures, whereby the middle layer, composed of poly (2-(dimethylamino) ethyl methacrylate) (PDMAEMA), condensed with miR-122 via electrostatic interactions. Delivery of miR-122 using this approach induced apoptosis and inhibited several of its downstream targets, including Bcl-w and the multi-drug resistance gene (MDR1), thereby reducing drug resistance. sCDP/DOX/miR-122 nanoparticles showed significantly enhanced anti-tumor potential compared to sCDP/DOX in vitro and in vivo.

#### 6.1.2. Synthetic Polymer-Based Nanocarriers

A variety of synthetic polymers, such as PLGA, PEI [140], polyurethane (PU) [141], poly(ε-caprolactone) (PCL) [142], and PLL, have been extensively explored as vehicles to carry therapeutic miRNAs [73]. Therapeutic inhibition of miR-21 in breast cancer cells was shown by using two different miR-21 inhibitors, miR-21 oligonucleotide (AMO) and miR-21 sponge plasmid DNA (Sponge), mediated by PLL-modified polyethyleneimine (PEI-PLL) copolymer nanocomplexes [143]. The study indicated that PEI-PLL/Sponge or PEI-PLL/AMO treatments in MCF-7 cells led to reduced miR-21 expression and cell viability.

*PLGA and PEG* have been approved by the FDA with regard to their safety and effectiveness [144]. PEGylation is known to enhance the stability and prolong the half-lives of the nanoparticles in circulation by reducing nanoparticle interaction with opsonins [145]. Combined PLGA–PEG nanoparticles were manufactured and conjugated with vitamin B12 to specifically deliver miR-532-3p mimics into CD320-overexpressed gastric cancer cells [146]. The therapeutic potential of a powerful synthetic polymeric nanocarrier system containing miR-34a mimic and volasertib, a small molecule polo-like kinase 1 (PLK1) inhibitor, was investigated for the treatment of PDAC [147]. PEG−poly[aspartamidoethyl(p-boronobenzyl)diethylammonium bromide] (PEG-B-PAEBEA) polymers self-assembled into micelles after electrostatic interaction with the miRNA and the small-molecule inhibitor. They markedly reduced tumor growth in orthotopic pancreatic tumor models in mice without any significant adverse systemic toxicity detected in major organs.

Dendrimers are a class of synthetic polymers with tree-like structures. These polymers are highly branched and uniform and have spherical geometry [148]. Dendrimers offer numerous advantages over traditional linear and branched polymers: (i) well-defined, adjustable nano-size range, (ii) capability to cross the cell membrane due to the controllable size of their globular morphology, and (iii) lipophilic properties and excellent flexibility that allow them to function as convenient drug- and gene-delivery systems, respectively, [149]. Moreover, dendrimers can be designed to have several functional groups, including NH_2_, OH, COOH, COONa, and CH_3_. Thus, with very simple surface modifications, dendrimers can be used as smart nanoparticles, and drugs, targeting moieties, or imaging probes can be readily encapsulated and conjugated owing to their good solubility and reactivity for specific attachment [81,149,150]. Dendrimer nanoparticles can be used not only to deliver drugs to specific sites but also to monitor the conditions of organs attacked by cancer cells and the progress of the treatment process [150]. Polyamidoamine (PAMAM) dendrimers, PLL dendrimers, peptide dendrimers, poly(l-lactide) dendrimers, PEG dendrimers, polypropyleneimine dendrimers, and poly(caprolactone) dendrimers are some of the various types of dendrimers. Among them, PAMAM dendrimers have been comprehensively studied as a nanocarrier system for the delivery of therapeutic genes and molecules [149,151]. PAMAM dendrimers were the first commercialized dendrimers. They are composed of hyperbranched polymers with repeating units of amide and amine [152]. PAMAM dendrimers are reasonably biodegradable, biocompatible, non-immunogenic, and have minimum non-specific blood–protein binding and controlled drug or gene release behavior, favoring their use as promising drug- and gene-delivery systems [149]. The positively charged amino groups in the internal cavities of PAMAM dendrimers interact with negatively charged nucleic acids to form dendriplexes, preserving the nucleic acids against degradation. It has been previously reported that PAMAM dendrimers can effectively transport DNA, siRNA, or miRNA to different cell types and exhibit a safer profile compared to other cationic polymers [153]. Tang et al. [154] fabricated a breast cancer-specific co-delivery system for 5-fluorouracil (5-FU) and miR-205 based on luteinizing hormone-releasing hormone (LHRH)-modified PAMAM. 5-FU and miR-205 interacted with functionalized PAMAM via electrostatic self-assembly and an amide condensation reaction, respectively. LHRH-targeted and miR-205- and 5-FU-co-loaded nanoparticles had a significant inhibitory effect on the proliferation, migration, and invasion of both MCF-7 and MDA-MB-231 cells in vitro and displayed better in vivo antitumor efficacy than their non-targeted counterparts. PAMAM dendrimer has been modified with chondroitin sulfate (CS), which is a tumor-targeted ligand for CD-44-overexpressed tumors used for miR-34a delivery [155]. Systemic delivery of a CS-PAMAM/miR-34a nanoformulation in mice bearing human lung adenocarcinoma cell line A549 xenografts exhibited successful delivery activity and favorable safety. Furthermore, a remarkable reduction in tumor growth and induction of apoptosis were also observed.

### 6.2. Inorganic Material-Based Nanocarriers for miRNA

A great number of inorganic nanocarriers based on gold, iron oxide, quantum dots, and silica have been proposed as miRNA vectors. These inorganic nanomaterials have been demonstrated to be promising non-viral gene-delivery systems. They are non-immunogenic, biocompatible, easily scalable, facile in functionalization, have thermal and chemical stability and low toxicity, and exhibit unique optical properties depending on their size and shape [156,157].

*Gold* is an inert material, which makes it suitable for use in living systems. Surface functionalization of gold with amine moieties facilitates the binding of negatively charged miRNA and protects it during blood stream circulation. Nucleic acids can also be thiolated to enable their direct attachment to gold via metal–sulfur bonds [80,158]. However, gold-based nanoparticles are notorious for their low storage stability, poor encapsulation efficiency, and slow endosomal escape [159]. Huang et al. [160] used gold in a nanocage formulation for combined applications of anti-miR-181b and photothermal therapies. Anti-miR-181b was successfully loaded onto PEI-modified, folate receptor (FR)-targeted, and PEG-decorated gold nanocages (AuNCs); the resulting nanocomplexes efficaciously enabled cellular uptake and reduced the viability of HCC cells. The study also demonstrated that AuNC-mediated delivery of anti-miR-181b, combined with laser irradiation, resulted in enhanced inhibition of tumor growth and apoptosis induction in HCC tumor-bearing nude mice. In a similar study, researchers designed novel AuNCs to co-deliver DOX and miR-122 mimic in combination with photothermal therapy (PTT) for HCC treatment [161]. Briefly, AuNCs modified with 11-mercaptoundecanoic acid (MUA) were loaded with DOX, whereas the gold surface was modified with PEI for miRNA attachment. Lastly, the nanocarriers were conjugated with PEG and HA to improve stability and targeting. The authors reported efficient in vitro and in vivo delivery of DOX and miR-122 using this approach. Moreover, this PPHAuNC–DOX/miR-122 multifunctional delivery system showed a better anti-tumor effect compared to any single treatment option, without causing significant side effects in the major organs, in a nude mouse model of HCC.

*Iron oxide*-based nanoparticles are another group of remarkable nanomaterials that have a wide variety of applications, including for hyperthermia, cell separation, imaging, and drug and oligonucleotide delivery [162]. They are effective targeting nanomaterials because they have superior superparamagnetic characteristics that help to guide them to accumulate in a specific tissue under an externally applied magnetic force [163]. Utilizing these nanoparticles as drug- or oligonucleotide-delivery agents generally involves surface modification with various polymers, such as PEI, PEG, PLL, albumin, sericin, chitosan, dextran, or lipids [164,165]. Many reports have demonstrated the potential of iron oxide-based nanoparticles in the delivery of RNA therapeutics against cancer in vitro and in vivo [166]. A multifunctional magnetic miRNA nanocarrier system using PEG-coated iron oxide nanoparticles was developed by Sun et al. [167]. Successful delivery of miR-16 to cells and tumor tissues of a mouse gastric cancer model using these nanoparticles was confirmed using multiple imaging modalities. miR-16-conjugated PEG-Fe_3_O_4_ nanoparticles remarkably reduced tumor growth and increased the sensitivity of gastric cancer cells to DOX. Sukumar et al. [168] co-loaded miR-100 mimic and anti-miR-21 onto β-cyclodextrin-chitosan (CD-CS) hybrid polymer-coated polyfunctional gold–iron oxide nanoparticles (polyGIONs) and then decorated them with GBM cell-targeting PEG-T7 peptide. This therapeutic nanocarrier system was intended to be used as an effective treatment for GBM, with nasal administration allowing it to reach the brain and bypass the blood–brain barrier. Intranasal delivery of polyGIONs led to considerable suppression of tumor growth in U87-MG GBM cell-derived orthotopic xenograft models in mice. The feasibility of this treatment modality was backed up by robust imaging, demonstrating the trafficking and intracranial tumor distribution of nanoparticles. Moreover, the survival rate of mice treated with polyGIONs in combination with the systemically delivered chemotherapeutic agent temozolomide (TMZ) was higher than that of the control and all of the single-treatment groups.

*Silica*-based nanocarriers hold great promise as they provide many benefits, such as having high loading efficiency and tunability and a readily functionalizable surface [169]. Silica nanoparticles (SiNPs) can be synthesized in different shapes, including as hollow, nanorod, dendritic, and mesoporous SiNPs [170]. In addition to drug-delivery applications, SiNPs have attracted much interest in recent years for the delivery of plasmid DNA, siRNA, and miRNA using their unique properties [82,170]. Li et al. [171] fabricated anti-miR-155-loaded mesoporous silica nanoparticles (MSNs) modified with polydopamine (PDA) and AS1411 aptamer (Apt) for targeted therapy of colorectal cancer (CRC). These nanoparticles successfully targeted CRC tumors and inhibited miR-155, resulting in tumor growth suppression, as well as sensitizing tumors to 5-FU chemotherapy.

### 6.3. Lipid-Based Nanocarriers for miRNA

Lipid nanoparticles have many advantages, such as high biocompatibility, biodegradability, tolerable toxicity, and reduced immunogenicity. These nanoparticles, which are formed by combining various lipids, differ in their physicochemical properties, such as their chemical compositions, sizes, charges, and particle surface characteristics. Developed as drug and nucleic acid carrier systems, lipid nanoparticles are commonly coated with polymers, which improve particle stability and half-life time in the circulatory system [172,173]. At present, many lipids with different characteristics (neutral, sterol, amphoteric, PEGylated, cationic, and ionizable) have been used to mediate the delivery of miRNA-based therapeutics.

Lipid nanoparticles have been widely examined, and they have successfully entered the clinic and received the first-ever FDA approval for the delivery of an siRNA drug, Onpattro [174]. There are also many in vitro and in vivo studies of lipid nanoparticles regarding the delivery of miRNA therapeutics. Wang et al. investigated the in vitro and in vivo therapeutic potential of cationic switchable lipid (CSL) nanocarriers conjugated with anti-GPC3 monoclonal antibody and co-loaded with sorafenib and miR-27a to achieve effective treatment of liver cancer [175]. In vivo studies in a liver cancer xenograft mouse model indicated that synergistic administration of sorafenib and miR-27a resulted in a threefold and 2.5-fold reduction in tumor size in comparison to control groups and free sorafenib-treated groups, respectively. The authors noted that CSLs maintain their stability in circulation, whereas they can change their molecular structure in the low pH environment of tumor cells and release their therapeutic payloads. Protonation of the central pyridine structure in these switchable lipids (by switching to intramolecular hydrogen bonding) causes a change in the hydrocarbon chain orientation of the compound, leading to the release of the therapeutic load from the nanoparticle [176]. In another work, co-delivery of melphalan and miR-181a from switchable lipid nanoparticles (LNs) enhanced therapeutic efficiency in retinoblastoma cells in vitro and in an in vivo rat model of xenografted retinoblastoma [177]. Quaternary amine−tertiary amine cationic lipid combination (QTsome) is a new-generation lipid nanoparticle formulation, obtained from a combination of quaternary amine-based cationic lipids (high charge) and tertiary amine-based cationic lipids (high pH responsiveness), that has a balanced charge and ideal pH behavior for oligonucleotide delivery. In general, the formation of QTsome from lipids such as 1,2-dioleoyl-3-trimethylammonium-propane chloride (DOTAP-cationic lipid) and 1,2-dioleyloxy-3-dimethylaminopropane (DODMA-ionizable lipid), which have previously been used in clinical trials, provides a great advantage for the of QTsome in the clinic. The Qtsome-AM-21 formulation, consisting of anti-miR-21 and several lipids, including DODMA, DOTAP, 1,2-dioleoyl-sn-glycero-3-phosphatidylcholine (DOPC-neutral lipid), cholesterol (Chol-neutral lipid), and N-(Carbonyl-methoxy-polyethylene glycol 2000)-1,2-dipalmitoylsn-glycero-3-phosphoethanolamine (DPPE-PEG), exhibited high colloidal stability and drug loading efficiency. Increased sensitivity to paclitaxel was observed in A549 cells treated with QTsome/AM-21 and decreased tumorigenesis was obtained in an A549 xenograft mouse model [178].

**Table 1:** Nanoparticles recently used for the in vivo delivery of miRNA mimics and inhibitors.

Material Type	Nanodelivery System	Conjugation	Targeting Ligand	Therapeutic miRNA	Synergistic Treatment	Target Genes	Delivery Route	Cancer Type	Ref.
Protein-based	Albumin-based nanoparticles	PLL	-	miR-329 mimic	-	eEF2K	IV	TNBC	[103]
Protein-based	Gelatin nanospheres	-	-	miR-506 mimic	-	PENK	Intratumoral injection	TNBC	[106]
Protein-based	Atelocollagen nanoparticles	-	RNA aptamer (APT)	miR-15a and miR-16-1 mimics	-	Bcl-2, cyclin D1, Wnt3a	-	Prostate cancer	[108]
Peptide-based	Chimeric peptide nanoparticles	Arginine	Plectin-1-targeting peptides (PTPs)	miR-212 mimic	DOX	USP9X	IV	PDAC	[113]
Peptide-based	Chimeric peptide nanoparticles	Arginine	Plectin-1-targeting peptides (PTPs)	miR-9 mimic	DOX	eIF5A2	IV	PDAC	[114]
Polysaccharide-based	Chitosan nanoparticles	L-arginine and Au nanoparticles	-	miR-125b mimic	Gefitinib	-	IV	Lung cancer	[124]
Polysaccharide-based	Chitosan nanoparticles	Conjugated linoleic acid (CLA)	Hyaluronic acid	miR-34a mimic	DOX	NOTCH, NF-κB	IV	Breast cancer	[125]
Polysaccharide-based	Dextran nanoparticles	Spermine	B1 receptor ligand	Anti-miR-21	-	PTEN	IV	Glioblastoma	[131]
Oligosaccharide-based	Cyclodextrin-based nanoparticles	PEI, Ad-CDM-PEG	-	miR-199a/b-3p mimics, anti-miR-10b	-	mTOR, PAK4, RHOC, EMT	IV	HCC	[138]
Oligosaccharide-based	Cyclodextrin-based star copolymer nanoparticles	PEG	-	miR-122 mimic	DOX	Bcl-w	IV	HCC	[139]
Synthetic polymer-based	PLGA	PEG	Vitamin B-12	miR-532-3p mimics	-	ARC	IV	Gastric cancer	[146]
Synthetic polymer-based	Poly[aspartamidoethyl(p-boronobenzyl)diethylammonium bromide] (PAEBEA)	PEG	-	miR-34a mimic	Volasertib	Bcl-2, c-myc	IV	PDAC	[147]
Synthetic polymer-based	PAMAM dendrimer	PEG	Luteinizing hormone-releasing hormone (LHRH)	miR-205 mimic	5-FU	-	IV	Breast cancer	[154]
Synthetic polymer-based	PAMAM dendrimer	-	Chondroitin sulfate (CS)	miR-34a mimic	-	Bcl-2	IV	Lung cancer	[155]
Inorganic-based	Gold nanocage	PEI, PEG	Folate receptor-targeted ligand	anti-miR-181b	Phototermal therapy	-	IV	HCC	[160]
Inorganic-based	Gold nanocage	PEI, PEG	Hyaluronic acid	miR-122 mimic	Doxorubicin Photothermal therapy	-	IV	HCC	[161]
Inorganic-based	Iron oxide-based nanoparticles	PEG	-	miR-16 mimic	DOX	Bcl-2	IV	Gastric cancer	[167]
Inorganic-based	Iron oxide-based nanoparticles	β-cyclodextrin-chitosan (CD-CS)	GBM cell-targeting T7 peptide	miR-100 mimic anti-miR-21	TMZ	PTEN, PDCD4	Intranasal delivery	Glioblastoma	[168]
Inorganic-based	Silica-based nanoparticles	PDA	AS1411 aptamer	anti-miR-155	5-fluorouracil	-	IV	Colorectal cancer	[171]
Lipid-based	Cationic switchable lipid nanoparticles	PEG	Anti-GPC3 antibody	anti-miR-27a	Sorafenib	FOXO1, PPAR-γ	IV	Hepatocellular carcinoma	[175]
Lipid-based	Cationic switchable lipid nanoparticles	PEG	-	miR-181a mimic	Melphalan	MAPK1, Bcl-2, BAX	Subcutaneous injection	Retinoblastoma	[177]
Lipid-based	QTsome nanoparticles	PEG	-	anti-miR-21	Paclitaxel	PTEN, DDAH1	IV	Lung cancer	[178]

## 7. Clinical Trials with miRNA Nanotherapeutics for Cancer

The first FDA approval for an siRNA-based therapeutic molecule in 2018 provided exciting hope for miRNA therapeutics. Even though miRNA-based therapeutics, including anti-miR compounds, specific miRNA inhibitors, and miRNA mimics, have recently been or are being investigated in preclinical, phase 1, or phase 2 clinical trials [179,180] for cancer and hepatitis C patients, the FDA has not yet approved their clinical use beyond investigational drugs. In this section, we focus on nanocarrier-mediated miRNA-based therapeutics in human clinical trials.

In 2013, the first miRNA-based drug, MRX34, which is a special amphoteric lipid nanocarrier formulation encapsulated in an miR-34a mimic, was tested in a phase 1 clinical trial (ClinicalTrials.gov identifier: NCT01829971). miR-34a was reported to have a crucial tumor suppressor role in various types of cancers by directly suppressing the expression of 30 oncogenes involved in several oncogenic processes, such as tumor growth, metastasis, and survival. Several groups reported that miR-34a is frequently lost or downregulated in patient tumors [181,182]. MRX34 was administered intravenously to 155 patients with several cancers, including primary liver cancer, lymphoma, and other solid tumors, biweekly over three weeks in four-week cycles. However, this phase 1 clinical study was terminated by the FDA due to multiple immune-related severe adverse events (SAEs), including fatal events. In 2016, MRX34 was investigated once again in melanoma patients but, due to the SAEs observed in the phase 1 study, the trial was withdrawn and future phase 2 trials were also terminated (ClinicalTrials.gov identifier: NCT02862145). Possible explanations for the failed miRNA-based therapeutics are that therapeutic miRNAs can also accumulate in healthy tissues as a result of the attachment of serum proteins on the surface of nanocarriers or because of interstitial fluid pressure and shearing stress promoting extravasation of nanocarriers, which may lead to their breakdown [183]. In the case of the MRX34 clinical trial, it is likely that the undesired toxicity problems arose from the payload, miR-34a, rather than the delivery system. This may also explain why the nanocarrier system used to deliver miR-34a failed to deliver the therapeutic doses to the tumors, as the miR-34a leaked into adjacent tissues, causing toxicity and even death.

The miRNA mimic drug TargomiR completed a phase 1 study and showed promising results in patients with recurrent malignant pleural mesothelioma or non-small cell lung cancer (ClinicalTrials.gov identifier: NCT02369198). The nanoformulation of TargomiR (miR-16 mimic) utilizes the EnGeneIC delivery vehicle (EDV), which is composed of non-living bacterial minicells, about 400 nm in size, coated with a cancer cell-targeting moiety: anti-EGFR bispecific antibody. Although some toxic effects have been observed in patients after miR-16 mimic treatment, this problem can be eliminated by optimizing the drug dose and applying an organ-specific approach [184]. This miRNA drug provides new hope for mesothelioma patients, who have less than 10% chance to reach 5 year survival [179]. A phase 2 study involving TargomiRs added to standard chemotherapy will be initiated soon [185].

## 8. Conclusions

Recently, there has been an explosion of research on miRNAs and miRNA-based therapeutics. A significant body of evidence suggests that miRNAs have significant potential for the development of targeted cancer therapeutics as they can specifically bind to and suppress the target gene. However, the performance of miRNA-based therapeutics in human trials largely depends on their safe and effective delivery to the target site at the desired concentrations. Nanocarrier systems have undoubtedly high leverage potential to overcome the challenges of in vivo miRNA delivery. These include enabling high stability while transiting through the systemic circulation, efficient miRNA loading, protection of miRNA from nucleases, endosomal escape, and cell-specific targeting. So far, reasonable efforts have been made to develop diverse polymer-, inorganic-, and lipid-based miRNA nanocarriers, and some have been translated into clinical trials. Several safer and more effective and clinically feasible novel nanocarrier formulations are being tested in preclinical and clinical settings. It has been projected that miRNA-based nanotherapeutics can be expected to be used in cancer treatment within the next 5 years.
